# Hand Sanitizer: Stopping the Spread of Infection at a Cost

**DOI:** 10.7759/cureus.61846

**Published:** 2024-06-06

**Authors:** Shreya Bhatt, Aasha Patel, Marc M Kesselman, Michelle L Demory

**Affiliations:** 1 Medicine, Dr. Kiran C. Patel College of Osteopathic Medicine, Nova Southeastern University, Davie, USA; 2 Dentistry, Roseman University College of Dental Medicine, South Jordan, USA; 3 Rheumatology, Dr. Kiran C. Patel College of Osteopathic Medicine, Nova Southeastern University, Davie, USA; 4 Microbiology and Immunology, Dr. Kiran C. Patel College of Allopathic Medicine, Nova Southeastern University, Fort Lauderdale, USA

**Keywords:** ocular manifestations of hand sanitizer, skin manifestations of hand sanitizer, gut-skin axis, covid-19, hand sanitizer

## Abstract

The recent rise in hand sanitizer use due to the COVID-19 pandemic has had a beneficial impact on stopping the spread of disease, but the potential negative implications of its overuse on the body and the microbiome have yet to be thoroughly reviewed. Epidermal layers absorb hand sanitizer from direct application to the skin, making them some of the most susceptible cells to the adverse effects of overuse. The increased usage of hand sanitizer can affect the variation, quantity, and diversity of the skin microflora, leading to conditions such as eczema, atopic dermatitis, and even systemic toxicity due to colonization of the skin with pathogenic bacteria. Due to the close-knit relationship between the skin and gut, the gastrointestinal system can also incur disruptions due to the negative effects on the skin as a result of excessive hand sanitizer use, leading to gut dysbiosis. Additionally, the accidental ingestion of hand sanitizer, and its abuse or misuse, can be toxic and lead to alcohol poisoning, which is an issue most commonly seen not only in the pediatric population but also in adolescents and adults due to aberrant recreational exposure. As a vulnerable body system, the eyes can also be negatively impacted by hand sanitizer misuse leading to chemical injury, visual impairment, and even blindness. In this review, we aim to highlight the variations in hand sanitizer formulation, the benefits, and how misuse or overuse may lead to adverse effects on the skin, gut, and eyes. In particular, we review the advantages and disadvantages of alcohol-based hand sanitizers (ABHSs) and non-alcohol-based hand sanitizers (NABHSs) and how the components and chemicals used in each can contribute to organ dysbiosis and systemic damage.

## Introduction and background

Since the start of the COVID-19 pandemic, there has been a drastic increase in hand sanitizer use. In February 2020, it was reported that there was a 73% increase in hand sanitizer sales in the United States compared to the previous year. The Centers for Disease Control and Prevention (CDC) and World Health Organization (WHO) recommended better and more thorough hand hygiene practices, and since then, hand sanitizers have been in high demand [1]. At one point during the pandemic, there was a severe shortage of sanitizers creating an uptick in do-it-yourself (DIY) sanitizers at home. However, to ensure safe use, DIY hand sanitizers should consist of the recommended amount of alcohol content, of at least 60% alcohol, as provided by the recommended CDC guidelines to ensure efficacy [2]. This raised the question of whether these formulations should be monitored further by the WHO and CDC, in an effort not to cause harm to those who use them. The CDC and WHO recommended the use of hand sanitizers during the pandemic to reduce pathogen spread when soap and water were not readily available [2], there can be more profound adverse superficial and systemic effects with the continuous and inappropriate use of alcohol-based hand sanitizers (ABHSs) and non-alcohol-based hand sanitizers (NABHSs). From the start of the COVID-19 pandemic, there was a 79% increase in average daily calls to poison control centers across the United States, compared to the previous two years, regarding toxic exposure to alcohol-based hand rubs or Lysol-like sanitizing aerosols, specifically in children [3,4]. The increased use of hand sanitizer led to questions about adverse and long-term effects. In this review, we highlight the current formulations of hand sanitizer as well as their benefits and limitations.

## Review

Formulations

**Figure 1:**
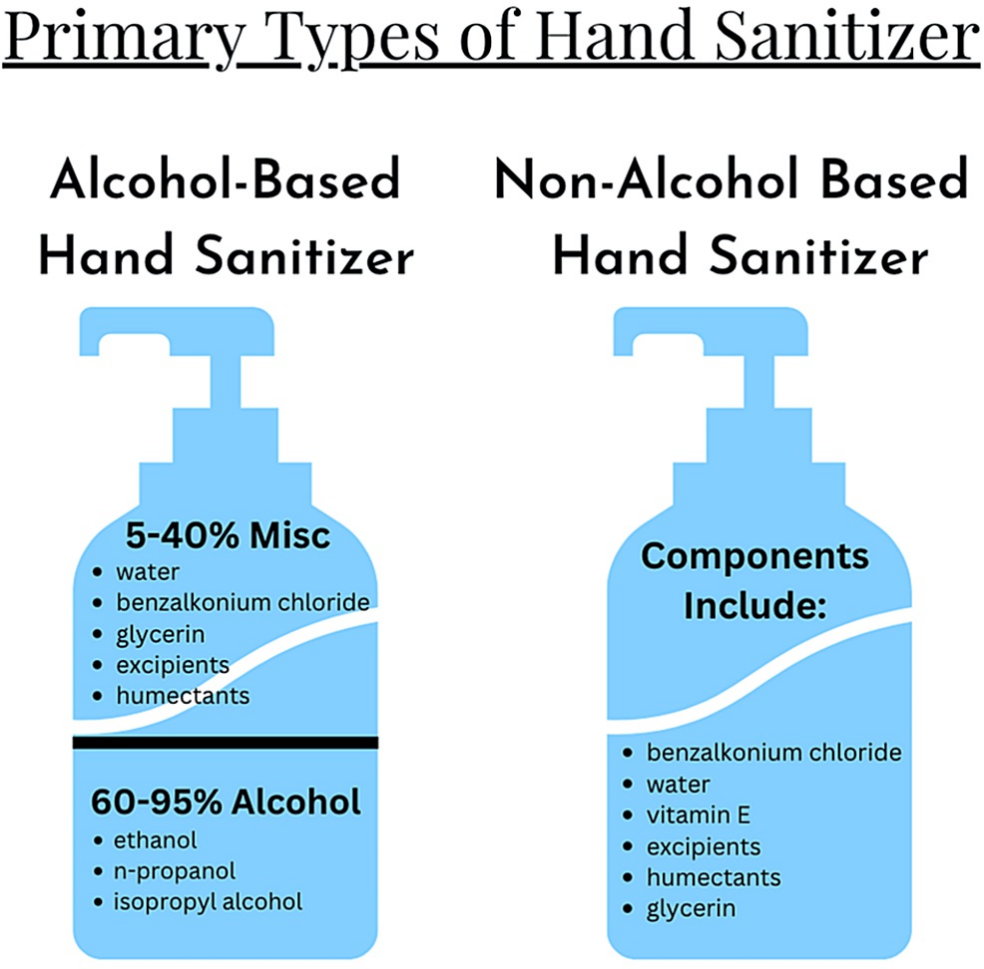
The two primary types of hand sanitizers and their respective components.

Among the various formulations of hand sanitizers, there are two primary categories: ABHSs and NABHSs. ABHSs are more commonly used, especially in healthcare settings, due to their advantageous qualities, including low cost, low volatility, minimal residual antimicrobial effects, and rapid time of action [5]. Several types of alcohols tend to be used in ABHSs, including ethanol, isopropyl alcohol, n-propanol, or a mixture of these as the active ingredients, along with water, excipients, such as fillers and viscosity agents like methylcellulose [6], and humectants [5]. Excipients are used to stabilize the product and increase the biocidal activity of the hand sanitizer by increasing the evaporation time of the alcohol, and humectants are often used to help avoid dehydration of the skin [5]. ABHSs and NABHSs with additive components also allow for an increased spectrum of activity and decrease the risk of resistance [7]. When these formulations are combined with other chemical components, they can also help lower concentration-dependent toxicity such that the negative effects of excess alcohol concentration in the hand sanitizer are decreased with the addition of excipients and humectants (Figure 1) [7].

According to the CDC, sanitizer formulations with 80% ethanol or 75% isopropyl alcohol, or sanitizers with 60%-95% alcohol, are considered effective and acceptable [8]. It has been found that 85% ethanol-based sanitizer with a 15-second contact time is sufficient in reducing Gram-positive and Gram-negative bacterial growth on agar plates. Despite this finding, it appears that ethanol and isopropyl alcohol are both efficacious [8,9]. At high alcohol concentrations of 85%-90%, there is the highest antimicrobial activity for both ethanol and isopropyl alcohol formulations and there are no statistically significant differences in their zones of inhibition [7]. At lower concentrations of 60%-100%, it appears that isopropyl alcohol has a wider range of inhibition zones than ethanol [7]. This result is likely because isopropyl has less of a minimal inhibitory concentration against most organisms compared to ethanol. Adding benzalkonium chloride (BC) as an excipient also enhances the activity of isopropyl alcohol and allows the hand sanitizer to have instantaneous and long-lasting effects [7]. Consumers often think that higher alcohol concentrations are always better. However, higher percentages of alcohol than recommended can dilute the necessary water content and can lead to the sanitizer being less potent, as proteins cannot be denatured without the presence of a certain amount of water [9].

**Figure 2:**
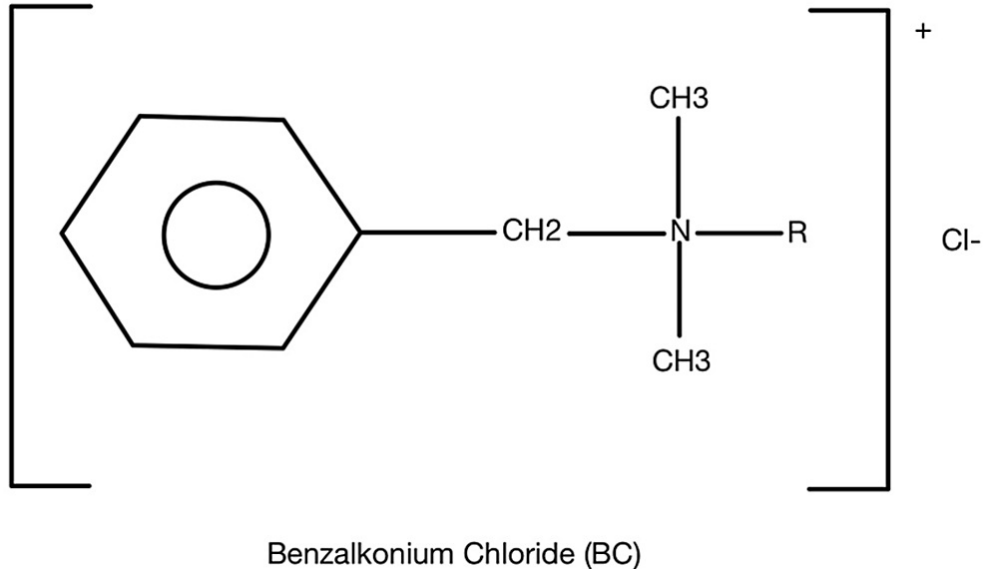
Chemical structure of benzalkonium chloride.

The main ingredient in NABHSs is BC, which is a quaternary ammonium (Figure 2). Hand sanitizers with this ingredient have been shown to be less irritating to the skin, compared to sanitizers that contain alcohol [5]. However, BC is not effective against non-enveloped viruses, with the exception of human coxsackie virus, but it is effective against gram-negative bacteria, fungi, and enveloped viruses [5]. This is because BC has various structural properties, including an alkyl chain tail that disrupts the bilayer of membranes as it can permeate the barrier and disturb the membrane’s physical and biochemical properties. This, in turn, interferes with protein function, and components of the bilayer are solubilized into BC micelles such that the membrane components are internalized and emulsified. BC has also been shown to break down intracellular targets including altering DNA conformation [5].

Additives that are introduced into alcohol and non-alcohol-based products also allow for an increased spectrum of activity while lowering the risk for resistance. When acting synergistically, these combinations can help lower concentration-dependent toxicity [7]. For example, glycerin can be added to sanitizer formulations to ease damage to the skin and prevent dryness. However, glycerin can also lower the antimicrobial activities of isopropyl alcohol, due to the reduced drug diffusion that comes with increased viscosity; therefore, this combination should be used with caution [7]. It has been found that the addition of BC to isopropyl alcohol systems improves the effectiveness of isopropyl alcohol. However, the use of BC alone has been proven to be more effective than when used in combination mixtures [7]. Ultimately, the various formulations of hand sanitizer can have a variety of effects on the body and its microbial activities depending on the ingredients.

In a study that tested the effectiveness of antiseptics, which is a topically applied chemical agent that decreases the microbial count and lowers the risk of infections [10], in reducing microbial load on the hands, alcohol formulations that have 70% alcohol were found to be the most effective to reduce rotavirus and Escherichia coli contamination on the hands compared to Savlon in tap water, liquid soap, and tap water alone [11]. However, despite the numerous advantages of hand sanitizer use including its efficient antimicrobial activity, immuno-protective qualities, and convenience, when used in excess, it can cause a multitude of adverse effects on a variety of bodily systems and their microbiomes, including the skin, gastrointestinal tract, and the eyes.

Skin

Human hands serve as a channel for the exchange of microorganisms between the body and the environment. Human hands can harbor pathogenic species, such as E. coli and methicillin-resistant Staphylococcus aureus, especially in healthcare settings where these bacteria are more prevalent [12]. Compared to a domestic setting, the use of ABHSs is particularly useful in these healthcare settings. ABHS has been shown, via skin sampling of sweat residues found in gloves, skin scrapings, or swab testing, to reduce the strictly pathogenic bacterial load on the hands and reduce the rates of infection at institutional locations, such as hospitals and schools [12]. The effectiveness of ABHS also appears to hold true within the general population [12,13].

The skin serves as a physical and immunological protective barrier that relies on an appropriately functioning epidermal microbiome, consisting of a wide variety of bacteria, fungi, viruses, micro-eukaryotes, archaea, and phages [14]. The efficacy of the skin’s barrier as well as its microbiome serve as a primary foundation for the skin’s immune system. If there is microbial dysbiosis of the skin, systemic pathological changes can result [15]. For example, microbial dysbiosis of the skin and gut can lead to inflammatory bowel disease (IBD), obesity, colorectal cancer, and allergic disorders [16,17]. Though there is still debate over which hand hygiene method is most beneficial and efficient at reducing skin pathogenic microbial load, it has been shown that the overuse of any hygiene products can lead to a disruption of the normal bacterial microflora of the skin, which can then negatively affect the skin and other body systems [16,17]. These effects can vary from colonization of the skin with pathogenic bacteria to eczema to systemic effects. 

Compared to skin on other body sites, the hand microbiome appears to be more dynamic and has a greater bacterial diversity. The commensal bacteria found on the hands include Actinobacteria, Bacteroidia, Flavobacteria, Sphingobacteria, Cyanobacteria, Bacilli, Clostridia, Fusobacteria, Alphaproteobacteria, Betaproteobacteria, and Gammaproteobacteria [18-22]. From self-reported data based on a study conducted in 2015, healthcare workers who utilized hand hygiene products more frequently than the general population was not found to have an increase or decrease in microbial diversity from the use of ABHSs or hand washing, unless the individual reported hand washing with soap and water over 40 times per shift [18-22]. In this instance, excessive hand washing was found to decrease the overall microbial diversity. This decrease in microbial diversity can have harmful effects because it can reduce the beneficial commensal bacterial populations making way for more opportunistic and pathogenic bacteria to colonize, dominate, and ultimately create an environment that could initiate or exacerbate disease [23]. However, these data do support positive and effective use of hand sanitizer when used in moderation.

Since this study in 2015, it has also been shown that the environment can heavily influence the diversity of bacteria on the skin. Based on a more recent study published in 2020, regarding the impact of urbanization and corresponding hygiene practices in South America, the increased use of cleaning and hygiene products, including chemicals utilized in hand sanitizer, decreased the microbial diversity at six human body sites, including the hands [24]. The data showed a reduction in the bacterial diversity of those participants in more urban areas. Interestingly, this study showed a change in the microbiome of the hand with the use of these chemicals, including a loss of many skin commensals that were replaced by Staphylococcus, Corynebacterium, Cutibacterium, and Micrococcus [24]. This study also found that the microbial profiles varied significantly among the various sampling locations, including the right arm, right hand, right foot, nasal, oral, and gut regions [24]. These data suggest that hygiene-related chemicals, including hand sanitizer, can alter the diversity of the skin microbiome in the various locations that were studied. 

Given the broad mechanism of action of hand sanitizer, particularly of alcohol-based varieties, pathogenic effects on the skin have been widely reported because of overuse. Skin dryness, associated with an increased amount of lipid-dissolving alcohols in hand sanitizer, can serve as an early sign of damage and dysfunction of the natural skin barrier. According to the WHO, ABHSs made primarily from ethanol, hydrogen peroxide, and isopropyl alcohol can become toxic to humans when misused [25,26]. Ultimately, excessive ABHS use can be associated with skin irritation, skin cracking, redness, and contact dermatitis [26]. This effect can be caused by lipid-emulsifying detergents, which serve to lower the number of lipids in the stratum corneum of the skin resulting in damage to the barrier function [27]. Diminished barrier function can allow allergens, irritants, and pathogens to cross the stratum corneum layer of the skin [28].

Studies have shown that overuse of hand sanitizers in healthcare workers correlates with the occurrence of hand eczema in those with a previous history of atopic eczema. A study from the Hubei Province in China showed that 321 out of 434 healthcare workers utilized hand sanitizer over 10 times per day, and 76.6% of these 321 workers showed signs and symptoms of irritant and allergic contact dermatitis [29]. Another study from Milan showed that in less than a two-month period in 2020, within the general population, 24 new cases of hand eczema were identified, and each case was related to the use of ABHSs. Many of these ABHSs were also found to be made with non-standard formulas that utilize methanol and isopropyl alcohol instead of ethanol, which could also play a contributing role to these pathologies [30]. These studies indicate that the rise of hand sanitizer overuse, which has increased in prevalence due to the COVID-19 pandemic, has been negatively affecting the general population and healthcare workers alike. The negative effects on the skin can also have a long-lasting detrimental impact on other parts of the body, including the gastrointestinal system.

Gut-skin axis and the gastrointestinal system

Anatomically, the skin and the gut both contain rich vasculature, which integrates into multiple body systems, such as the immune and endocrine systems [31]. Due to the interconnected features between the integumentary and gastrointestinal systems, many gastrointestinal disorders often present with cutaneous manifestations such as atopic dermatitis, psoriasis, acne vulgaris, eczema, dandruff, and possibly even skin cancer [32-36]. The gastrointestinal microbiota can create neurotransmitters, metabolites, and hormones, via the influence of the diet or directly, which can modify the skin upon entering the circulation [31]. The inherent interconnectedness of the gastrointestinal system and the skin serves as an indication that the detrimental effects of hand sanitizer overuse may also have greater implications on the gut-skin axis, especially in cases of accidental hand sanitizer ingestion. As the gastrointestinal system also serves as one of the body’s primary interfaces with the external environment, the gut plays a key role in maintaining body homeostasis, which can be perturbed by the overuse of hand sanitizer and can lead to gut dysbiosis [31,37].

Due to the integrated relationship between both body systems, dysbiosis resulting from diseases such as IBD can cause toxins and bacteria to escape from the gut through a leaky gut barrier, and if these are not appropriately processed by the liver, the skin can be affected due to the creation of a pro-inflammatory environment [31]. If the intestinal barrier is disrupted due to a leaky gut or gap areas, it has also been found that pathogenic bacteria and microbial metabolites along with intestinal microbiota can enter the bloodstream and accumulate in the skin causing a perturbation of the skin’s homeostasis, barrier integrity, and disturbed differentiation of the epidermis [31,38,39]. High levels of metabolites can even decrease the hydration of the skin and impair keratinization [38,39]. For example, studies have shown that intestinal dysbiosis can also be linked to atopic diseases such as atopic dermatitis. When the intestinal barrier is dysfunctional, this can contribute to the absorption and penetration of undigested food, toxins, and microbes into the circulation, which can cause the T-helper 2 (Th2) cell-mediated immune response to be activated resulting in even more tissue damage to the skin [31,40-44].

Intestinal dysbiosis is also linked to atopic dermatitis [44]. After collecting fecal samples from patients with the atopic disease, Faecalibacterium prausnitzii bacteria levels are significantly decreased in comparison to the control group alongside a decrease in short-chain fatty acid production [43,45]. This led to the conclusion that there may be a positive feedback loop, secondary to uninhibited inflammation of the epithelium, involving intestinal dysbiosis with respect to F. prausnitzii and disruption of the epithelial barrier.

Similar to how the gut affects the skin, the skin also reciprocally influences the gut. An example of this is the link between the gut microbiome and atopic dermatitis. It has been found that the incidence of atopic dermatitis is higher in more developed countries, indicating that excessive hygiene practices, such as the overuse of hand sanitizer, may impact the body’s microbiota and prevent the body from adapting useful immune responses to potentially dangerous pathogens. In cases of atopic dermatitis, gene defects cause Th2-mediated as well as physical disruptions to the skin barrier leading to an increased risk of infection and allergic reactions. This barrier is further perturbed by the increase in scratching of the pruritic, dry areas of the skin affected by this condition. Eventually, the microbial milieu of the skin is altered resulting in a diseased state. Multiple prospective studies have shown that the colonic microbiomes of infants are less diverse in those that develop atopic dermatitis, indicating that an irregular gut microbiome is associated with atopic dermatitis [46]. This finding suggests that in comparison to the way an increase in diversity of the microbiome throughout the body is beneficial in the promotion of gut health, drier skin may create a more diverse microbiome, which can be harmful and lead to disease and other pathologic conditions if this increased diversity leads to an impaired skin barrier.

More specifically with respect to hand sanitizer, since the COVID-19 pandemic, sales of hand sanitizer products have risen by 838% [47]. Keeping this percentage in mind, some concerns have risen regarding gut health. Maintaining a healthy gut microbiome can positively affect one’s overall health, especially immune health [36,48-50]. There are beneficial microbes in the gut that can act as defense mechanisms against toxins or pathogens, however, these microbial colonies are delicate, and they can be diminished by many small factors, such as allergies, obesity, and other alterations to gut flora [36,48-50]. Some components in the antimicrobials of consumer products can lead to health risks and environmental risk factors for IBD. Triclosan (TCS), triclocarban (TCC), BC, benzethonium chloride (BET), and chloroxylenol (PCMX) are all common ingredients in cleaning products [51]. Mouse models have been used in a limited number of studies to determine the adverse effects of hand sanitizer and antimicrobial chemicals. BC was found to increase dextran sodium sulfate (DSS)-induced colonic inflammation and colon tumorigenesis in mice and increase Toll-like receptor 4 signaling activation by disturbing the barrier function of the intestines and ultimately elevating the circulating levels of bacterial products [52]. In a study conducted on mice, exposure to TCC increased the amount of the pro-inflammatory bacteria, Proteobacteria, which is found to have greater concentrations in patients with IBD, and decreased the amount of the anti-inflammatory bacteria, Bifidobacterium [51]. These findings indicated that the expansive use of antimicrobial compounds, including this primary ingredient used in many hand sanitizers, could exacerbate the development of diseases that disrupt gut microbiota including IBD and colon cancer [51].

In 2016, the FDA banned TCS and TCC from all over-the-counter hand-washing products in the United States, as they have been shown to have adverse effects on gut health such as colonic inflammation. Although there is not much evidence of chronic exposure to these chemicals on overall health, there is evidence that even low-dose consumer antimicrobials can affect gut health [51]. Although it is banned in the United States, TCS is still a common ingredient restricted to smaller quantities in household products and hand sanitizers used in other countries. This chemical can be absorbed through the skin and enter the bloodstream [53]. With the recent pandemic, exposure to TCS has increased along with the increased use of detergents and household antimicrobials. TCS is an endocrine-disrupting chemical that can have a negative impact on the gut microbiome by harming the compositional and functional levels of the gut flora. After 13 weeks of TCS exposure in mice, there were significant differences in the microbial communities and shifts in the bacterial families of the gut microbiome in the treatment versus control groups of the mice. Because healthy gut flora is a key factor in maintaining overall health, extensive exposure to TCS can lead to severe illnesses, such as endocrine disorders, antibiotic resistance, colonic inflammation, and colonic tumorigenesis. In addition, extensive exposure in early childhood can cause disturbances in metabolism and gut microbiota, which can affect a child’s life in the future and worsen over time [53]. Overall, while TCS is no longer used in the United States, its effects, particularly when used in excessive amounts or frequencies, are significant to individuals in other countries.

With the rise in household hygiene products during the pandemic, it is possible that the negative effects of hand sanitizer overuse could also be seen with excessive use of these products. Domestic hygiene products often include a vast amount of chemicals that can negatively impact the human gut microbiome and host health, especially upon accidental ingestion [54]. A study was conducted on thirteen young, healthy adults who donated one fecal sample. Within these stool samples a variety of food additives and household hygiene products, such as dishwasher detergent, were tested to see their impact on various components of the microbiome in treatment versus control groups. It was found that dishwashing detergent caused a significant decrease in gut microbiome diversity, and there was also a significant decrease in total bacteria compared to the control group. However, dishwashing detergent was found to increase the concentration of E. coli which could have pathogenic effects [54]. In 2019, a study published in the European Journal of Nutrition showed that gut microbiome diversity decreased with the regular use of dishwasher detergent. With the decrease in gut microbiome diversity, there was evidence of a decrease in beneficial microbial metabolites found in normal gut flora, such as short-chain fatty acids. The absence of short-chain fatty acids can lead to cellular damage of the gut wall, ultimately, leading to gut inflammation [54]. This resulting gut inflammation is similar to the gut inflammation caused by BC, a primary component in NABHSs. These findings, therefore, suggest that if gut microbiome diversity is being diminished by household products such as dishwasher detergent, hand sanitizers may also be causing similar damage, although additional research is required to confirm this hypothesis.

Additionally, the improper use of ABHSs can have a negative impact specifically on pediatric populations as ingesting more than a couple of mouthfuls of ethanol-based hand sanitizer can lead to alcohol poisoning. According to Gold, et al. and the United States National Poison Data System, there have been reports of 65,000 incidences of ethanol-based hand sanitizer ingestion between 2011 and 2014 [9]. Multiple studies have also found that the ingestion of ethanol from hand sanitizers can lead to intoxication and hypoglycemia in children [9]. Older children have also been found to recreationally ingest hand sanitizer as a means of becoming inebriated [9]. Outside of the obvious negative effects of alcohol ingestion especially via the means of hand sanitizer, there are greater implications with hand sanitizer overuse that can ultimately create gut dysbiosis, cause inflammation, and even lead to colon cancer [51].

Ocular

The excessive use of hand sanitizer can also have toxic effects on the ocular region [4]. Chemical exposure from hand sanitizer in the eye can lead to an elevated risk of toxicity as well as pathophysiological damage, especially among young children [4]. Recent studies showed that sustained exposure to ABHSs resulted in chemical injury to the ocular region [55]. More specifically, chemical burns can result from the alcohol content of the sanitizer when it encounters ocular tissue, such as corneal, limbal, or conjunctival tissues [56]. The alcohol content in the sanitizers can also have a negative impact on the physiological functions of the eye by leading to a reduction in the proliferative capacity of cells, inducing apoptosis of human corneal limbal epithelial cells, and reducing mucosal immune response, especially on the ocular surface [57]. Previous studies have reported that a 50% or higher concentration of ethanol in solution can cause loss of corneal epithelial cells and stromal keratocytes, leading to corneal inflammation and edema [58]. In addition, 62% of gel-type ethanol sanitizers have led to extensive defects in the corneal and conjunctival epithelial cells as well as damage in the limbal stem cells. A week after treatment was initiated, there was evidence of long-term limbal cell deficiency due to prolonged epithelial defect, supporting the idea that long-term effects of prolonged ocular chemical burns due to exposure to the ethanol within hand sanitizers are a true ocular emergency, as damage may lead to permanent visual morbidity [55]. 

Analogous to gel hand sanitizers, aerosol hand sanitizers can also be harmful to the ocular region. The amount of exposure and frequency of usage of aerosol alcohol-based sanitizers is proportional to the severity of injury. The severity of the injury is concentration-dependent, and this can pose a great threat to the eyes [55,59]. Ocular surface discomfort and precorneal tear films have been reported when inspecting the adverse effects of indoor aerosolized sanitizers [60,61]. In addition, alcohol is known to have dehydrating properties, therefore, if in contact with the ocular mucosa, it can result in stress or damage to cells and tissues, leading to symptoms of dry eye disease [62]. Similar to gel hand sanitizers, aerosol sanitizers can cause stress on ocular cells, leading to an increase in the level of inflammatory factors in the ocular surface epithelium. The amplified inflammatory response serves as an itch stimulus, further causing irritation [57]. To treat this irritation, immediate irrigation is the most common and effective method; however, more serious cases require antibiotic treatment and topical steroid eye drops [55]. Ultimately, when used in excess or irresponsibly, aerosolized hand sanitizers and ethanol gel sanitizers can have harmful and long-term negative effects on the eyes and must be used sparingly and with caution.

Discussion

With the drastic increase in hand sanitizer consumption since the start of the COVID-19 pandemic, it has become paramount now more than ever to better understand the implications of excessive use of potentially toxic chemicals. Hand sanitizer can come in alcohol-based and non-alcohol-based formulations and when used in appropriate quantities, can have immense benefits with respect to hygiene and protection from various pathogens. However, when used in excess, the effects could have wide-ranging consequences including microbial resistance and organ system damage.

The increased use of hand sanitizer poses the risk of increasing antimicrobial resistance. This has been occurring to such a vast extent that many COVID-19 patients are also being treated with antibiotics to reduce the risk of secondary bacterial infections [63]. It has been found that bacteria can develop resistance to certain cleaning products and chemicals when they are exposed to a lower concentration of the product multiple times. However, the appropriate use of hand sanitizer, in which an individual properly cleans their hands for 20-30 seconds, can keep bacteria from becoming resistant [63]. Irregular and diluted usage of cleaning chemicals outside of hand sanitizer has also been shown to allow resistant bacterial strains to survive resulting in an overall resistance among microbes [63]. Antimicrobial resistance has been found to result in over 700,000 deaths around the world every year, so it is vital to use hand sanitizer appropriately to avoid perpetuating this growing issue. Based on a study conducted by Pidot in 2018, the bacteria Enterococcus faecium, which is a leading cause of hospital-acquired infections is becoming resistant to ABHSs [63,64]. Thus, the appropriate use of hand sanitizer is essential in avoiding antimicrobial resistance.

Excessive hand sanitizer use can disrupt the skin and gut microbiomes and a lack of skin microbial diversity has been associated with atopic dermatitis, skin cancer, psoriasis, dandruff, and acne vulgaris [65]. In addition, studies have shown that an imbalance of beneficial and pathological bacteria within the gut microbiome can play a significant role in IBD, irritable bowel syndrome, diabetes, obesity, cancer, and cardiovascular and central nervous system disorders [17]. Therefore, it seems possible that future studies regarding the COVID-19 pandemic will likely find that excessive hand sanitizer use can result in similar pathophysiological conditions. As described above, the inappropriate use of hand sanitizer can also lead to ocular pathologies and visual impairment. Recent findings have also shown that gut dysbiosis can have a strong influence on various ocular conditions and can contribute to the progression of diseases like uveitis, dry eye syndrome, glaucoma, and macular degeneration [66].

Outside of hand sanitizer effects on the skin, gut, and eyes, there has even been evidence of a connection between the oral cavity and hand microbiome, suggesting that excessive hand sanitizer use can create dysbiosis in the mouth as well [67]. While very few studies have been published to date examining the effect of hand sanitizer use on the oral microbiome, given the frequency with which people touch their hands to their mouth, it is possible that excessive hand sanitizer use could disrupt the abundancy or diversity of the oral microbiome. Oral dysbiosis has been linked to dental caries, osteoporosis, periodontitis, and oral cancer. These conditions can lead to bone fractures, alveolar bone, and periodontal ligament loss, and can affect the functionality of other organ systems [68]. Future studies should examine the impact of excessive hand sanitizer use on the bacterial diversity of the oral microbiome and downstream pathologic effects. Overall, microbiome dysbiosis can result in a variety of pathologies in numerous organ systems, and the overuse of hand sanitizer may contribute to the exacerbation of these conditions.

Based on our review, there are still certain limitations that require further research, including a specific definition of “excessive use” of hand sanitizers worldwide. The term can be interpreted in various ways, as there was not a set numerical definition available that was consistent among the various studies that have been conducted. In addition, there was a lack of data on the direct effects of hand sanitizer on various organ systems, such as the respiratory or oral systems, and how their microbiome may also be altered with the increased use of hand sanitizer. Despite the limitations faced, there is still strong evidence that any defined overuse of hand sanitizer can lead to dysbiosis and ultimately contribute to various health-related adversities.

## Conclusions

While there is an increasing prevalence of hand sanitizer use around the world, there is still a lot that is unknown about the effects of excess hand sanitizer use on various organ systems. In this study, we review the limited research available focusing on the harmful effects of hand sanitizer overuse on the skin, gastrointestinal system, and ocular region. However, further research must be conducted to appropriately assess the effects of hand sanitizer overuse on additional organ systems as well as the potentially harmful bodily effects of other commonly utilized domestic hygiene products that may share common formulation ingredients. Further investigation can ultimately ensure that the health and well-being of society are not compromised in light of this long-standing global pandemic.
